# Predicting the vulnerability of birds to trophic threat posed by phenological mismatch based on nutritional and physiological status of nestlings

**DOI:** 10.1093/conphys/coz096

**Published:** 2019-12-06

**Authors:** Shuping Zhang, Lidan Zhao, Xinjie Zhang, Wei Liang

**Affiliations:** 1 College of Life and Environment Sciences, Minzu University of China, Zhongguancun south street 27, Beijing 100081, China; 2 Ministry of Education Key Laboratory for Ecology of Tropical Islands, College of Life Sciences, Hainan Normal University, Longkun south road 99, Haikou 571158, China

**Keywords:** birds, nutritional quality, phenological mismatch, trophic threat, vulnerability

## Abstract

Climate change induced phenological mismatches between nestlings and their optimal food resources have been found to negatively influence the survival of many bird species. Discriminating which species is vulnerable to such threat is difficult only based on the diet observation, and therefore it is necessary to establish a more reliable method to predict the vulnerability of bird species. In the case of Asian short-toed lark (*Calandrella cheleensis*), we predicted such vulnerability by evaluating whether nestlings can absorb equal level of nutrients from different diets and maintain equal physiological status. We compared the diet, plasma nutrients, plasma insulin-like growth factor-1 (IGF-1), body mass and survival rate of nestlings hatched under different optimal food (grasshopper nymph) abundance conditions in two breeding seasons. Plasma glucides, amino acids, tricarboxylic acid (TCA) cycle metabolites, some fatty acids, IGF-1, body mass and survival rate of the nestlings hatched under medium or low nymph abundance conditions were significantly lower than those of nestlings hatched under high nymph abundance condition. The relative abundance of plasma amino acids, glucides, TCA cycle metabolites and fatty acids were significantly, and positively, correlated with IGF-1 levels, which, in turn, was positively correlated with nestling body mass. These results indicate that the diet with low optimal food proportion was nutritionally inferior to the diet with high optimal food proportion and inhibited the growth of nestlings. Species like Asian short-toed lark is vulnerable to the trophic threat induced by phenological mismatch because the alternative food is insufficient to satisfy the nutritional requirement of nestlings.

## Teaser

Lay summaries:

The vulnerability of bird species to trophic threat posed by phenological mismatch should be predicted on the basis of nestling’s nutritional and physiological status rather than only on diet observation. The species is vulnerable to such threat when the alternative food is insufficient to satisfy the nutritional requirement of nestlings.

## Introduction

In temperate environments, high-quality foods suitable for nestlings are typically only available within a short time window each year, which means that insectivorous birds must time their breeding, so that their nestlings hatch within this window (Naef-Daenzer and Keller, 1999). The breeding performance of insectivorous birds is often dictated by the degree to which parents can synchronize the peak of offspring food requirement with the peak abundance of insect prey (Naef-Daenzer and Keller, 1999; Both *et al*., 2006). Climate change can cause different degrees of phenological mismatch between nestlings and insect food resources, resulting in asynchrony between peak nestling food requirement and that of insect food abundance. In the past 20 years, numerous such phenological mismatches have been found between nestlings and insect food resources in many bird species (Visser *et al*., 1998; Visser and Both, 2005; Reed, 2013; Burgess *et al*. 2018). Bird species that can adjust nestling hatching dates to track shifting peaks of optimal food abundance can avoid, or at least minimise, phenological mismatches, but those cannot do this could suffer the trophic threat and population declines (García-Navas and Sanz, 2011; McKinnon *et al*., 2012; Burger *et al*., 2012; van Gils *et al*., 2016).

Predicting which species are vulnerable to such trophic threat is crucial to understand the effect of climate change on the viability of bird species. The available studies show that most of bird species have limited ability to adjust the timing of hatching in response to temporal changes in the peak abundance food resources (Visser *et al*., 1998, Ovaskainen *et al*., 2013, Doiron *et al*., 2015, Zhang *et al*., 2017). The option available to such species is to adjust their nestlings’ diets when the phenology mismatch happens. Some researchers have found that birds may adjust their nestling’s diets if their optimal food is not sufficiently abundant (Dunn and Møller, 2014; Vatka *et al*., 2016). However, other studies have found a positive relationship between low dietary diversity and higher breeding success at the population level, which has been explained by a higher consumption of optimal prey in those populations with lower diet diversity (García-Navas and Sanz, 2011; Resano-Mayor *et al*., 2014). Bird species are likely to vary in their vulnerability to the trophic threat of phenological mismatches. It is urgent to assess such vulnerability for more species in order to provide information for conservation.

Although many studies have found that adult birds provide alternative foods to nestlings when optimal foods are less abundant (Naef-Daenzer *et al*., 2000; García-Navas and Sanz, 2011; Burger *et al*., 2012; McKinnon *et al*., 2012; Samplonius *et al*., 2016; Vataka *et al*., 2016), few studies can clarify whether the nutritional quality of the alternative is equal to the optimal food, or whether the alternative food can functionally replace the optimal food. This makes it difficult to determine the vulnerability of a bird species to phenological mismatch from observational data alone. Nutrients assimilated into the blood from food are, however, direct indicators of the nutritional quality of ingested food (Reynolds *et al*., 2003; Arnold *et al*., 2010). Plasma metabolite levels have been found to be reliable predictors of both changes in the individual body mass of adult birds (Jenni-Eiermann and Jenni, 1994; Guglielmo *et al*., 2005; Cerasale and Guglielmo, 2006; Dietz *et al*., 2009) and the body condition and growth rates of nestlings (e.g. Villegas *et al*., 2002; Nadolski *et al*., 2006; Amat *et al*., 2007; Albano *et al*., 2011). Therefore, to determine the actual impact of an apparent trophic mismatch on nestlings requires directly assessing their blood nutrient level and physiological status.

The physiological pathway connecting diet and body condition has been well documented in vertebrates (Lupu *et al*., 2001; Stratikopoulos *et al*., 2008; Lodjak *et al*., 2014). In brief, nutritional compounds (e.g. amino acids) in the blood are indicators of the synthesis of the most important growth related hormones, “insulin-like growth factors (IGFs)”, which can alter gene transcription (Fingar and Blenis, 2004; Laviola *et al*., 2007), stimulate protein synthesis (Nakae *et al*., 2001; Stratikopoulos *et al*., 2008), cell growth and differentiation (O’Kusky *et al*., 2000; Lupu *et al*., 2001), thereby increasing muscle mass and bone-tissue remodelling (Yakar *et al*., 2002; Lodjak *et al*., 2016). Nutrients assimilated into blood from food positively determine the body condition of the nestlings via this pathway. Therefore, IGF-1 levels can be used to assess whether alternative foods are nutritionally equivalent to optimal foods. Nowadays, metabolomic analysis has become a powerful technique for measuring and comparing plasma compounds under different conditions (Colinet *et al*., 2012). Investigating the plasma metabolomes of nestlings fed different diets can provide comprehensive blood nutrient and nutrition-related metabolic data for assessing the nutritional value of each diet. In other words, the blood metabolomes, IGF-1 and body mass, of nestlings can be used as indicators to determine whether the alternative diet is nutritionally and physiologically equivalent to the optimal diet.

In this article, we predicted vulnerability of bird species to the trophic threat induced by phenological mismatch according to evaluating whether nestlings can absorb equal level of nutrients from different diets and maintain equal physiological status in the case of the Asian short-toed lark (*Calandrella cheleensis*), a species with a clearly defined optimal food, grasshopper nymphs. We hypothesised that parents would adjust their nestlings’ diet when their nestling’s hatch date did not coincide with the peak of optimal food abundance. Based on the physiological relationship between diet and body condition, we also hypothesised that the levels of plasma metabolites, IGF-1, body mass and survival rate of nestlings provided with alternative foods would be lower than those of nestlings that were predominantly fed the optimal food. To test these hypotheses, we compared nestling diet in different optimal food conditions and compared nutritional and physiological indicators, body condition and survival rate of nestlings fed different diet. If the results support our hypothesis, we predict the species is vulnerable to the threat.

## Materials and methods

### Study site and species

Our study area was in the Hulun Lake National Nature Reserve (47°45′50″N–49°20′20″N ; 116°50′10″E–118°10′10″E), which is in the northeastern part of the Inner Mongolian Autonomous Region, China. This is a semiarid, steppe region where the mean annual temperature, precipitation and potential evaporation are −0.6°C, 283 and 1754 mm, respectively. The dominant plant species are *Stipa krylovii*, *Leymus chinesis* and *Cleistogenes squarrosa*. Winter is longer than summer and the approximate average maximum daytime temperatures in January and July are −20.02 and 22.72°C, respectively.

The Asian short-toed lark is the most common passerine species living on the grasslands of northeastern Inner Mongolia of China (Tian *et al*., 2015; Zhang *et al*., 2017). It is an ideal species on which to test our hypotheses because it only raises one brood per season and the grasshopper (Orthoptera) nymph is the main component (about 80%) of the nestlings’ diet (Tian *et al*., 2015). There is only one significant nymph abundance peak once a year in the study area (Chen and Gong, 2005; Guo *et al*., 2009). The breeding of the species begins in mid-April and end in mid-June. The average clutch size is 3.05 ± 0.51, and the nestlings remain in nests 8 days (Tian *et al*., 2015).

### Field-data collection

**Figure 1:**
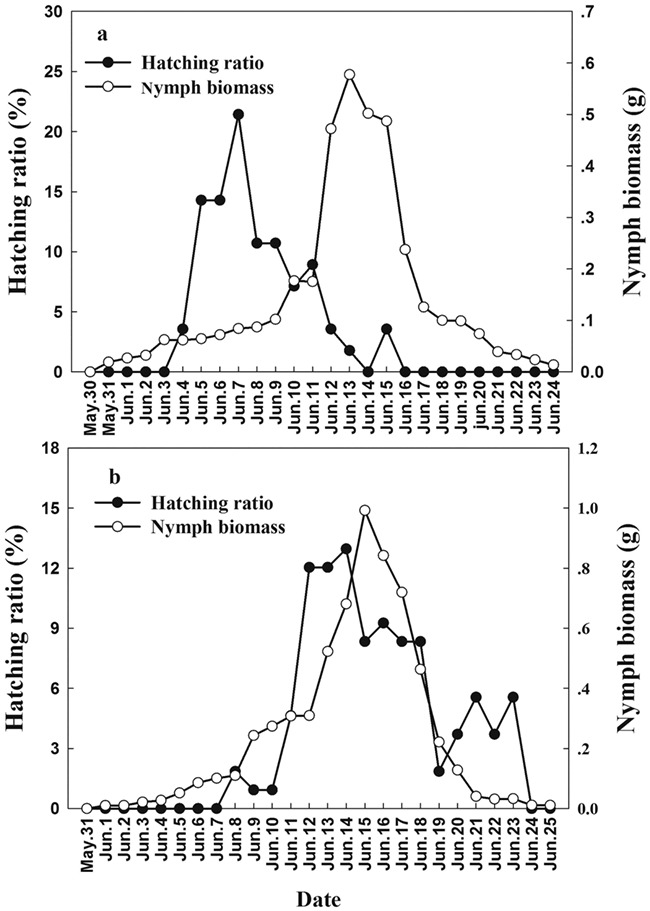
The hatching ratio of Asian short toed lark and grasshopper nymph biomass at Hulun lake nature reserve in 2016 (a) and 2017(b). Hatching ratio is the number of nests with newly hatched nestlings divided by the total number of monitored nests. Nymph biomass was the mean nymph biomass collected on 10 transects per sampling day.

We monitored Asian short-toed lark nests in the study area daily from 20 April to 27 June in 2016 and 2017, in order to record hatching dates, nestling diet, nestling body mass, the numbers of nestlings hatched, the number of nestlings surviving to fledging and collect nestling blood samples. These data were obtained from 84 and 72 nestlings (one nestling per nest) in 2016 and 2017, respectively. Any eggs and nestlings that disappeared, presumably due to predation, were excluded from the analysis. Because nestlings remain in the nest for 8 days, we used the body mass of 7 days old nestlings as a measure of nestling body condition. The diets of 4-days-old nestlings from different nests were quantified using the neck-collar method (Moreby and Stoate, 2000). Nestlings were fitted with neck-collars made from braided cotton thread at 8:00 and 18:00, left for 30–40 minutes, after which any food items contained in their throats were carefully extracted and the collars removed. Our previous observations of nestling diet indicate that the grasshopper nymphs are the optimal food (Tian *et al*., 2015). The relative abundance of grasshopper nymphs in the study area was quantified by catching these in an insect net on 10 parallel, 2 × 100 m sampling transects, spaced 10 m apart, every 2 days. Captured nymphs were dried and weighed to determine their biomass. The mean daily nymph biomass was the average daily biomass obtained from all 10 transects. The annual hatching ratio and nymph biomass variations in 2016 and 2017 are shown in Fig. 1.

### Nestling blood samples

About 100 μl of whole blood was collected from 4-days-old nestlings at 14:00 of the sample day to measure plasma metabolome and IGF-1 levels. A brachial wing vein of each nestling was punctured with a disinfected 23 G needle within 1–3 minutes of capture and blood that exuded from the puncture site was collected into heparinized microcapillary tubes. The skin around the puncture site was disinfected with medical alcohol before and after puncturing. Pressure was applied to the puncture site for 1 minute with an alcohol-soaked cotton wool swab to staunch bleeding. Blood samples were centrifuged at 4000 rpm for 20 minutes. The resultant plasma was snap frozen in liquid nitrogen and stored at −80°C until required for metabolite and IGF-1 assays. Blood sampling procedures complied with ARRIVE guidelines. Permission to handle our study animals was given by Hulun Lake National Reserve Administration and Animal Research Ethics Committee of the Hainan Provincial Education Centre for Ecology and Environment, Hainan Normal University (permit no. HNECEE-2014-005).

### Metabolomic analysis using GC–MS

Plasma metabolites were analysed with gas chromatography–mass spectrometry (GC–MS). Methods were as follows:

#### Serum preparation

After thawing at 4°C for 30 minutes, 50 μL of diluted plasma (1:5) was added to 200 μL of methanol containing the internal standards (5 μg/ml of succinic acid-d4) and vortexed for 30 seconds. The solution was then centrifuged at 14 000 rpm for 15 minutes at 4°C after which the supernatant was removed and lyophilized. The residue was re-dissolved in 50 μL of pyridine (containing 20 mg/ml methoxyamine). After being vortexed and ultrasound-treated, an oximation reaction was performed in a water bath for 1.5 hours at 37°C. This was followed by a silylation reaction conducted with 40 μL of MSTFA in a water bath for 1 h at 37°C. After centrifugation at 14 000 rpm for 15 minutes at 4°C, the supernatant was analysed with GC–MS. Quality control (QC) samples were prepared by taking 20 μL of serum from each sample for evaluation. Six QC samples from each year were analysed in parallel to evaluate their repeatability. QC samples were prepared according to the same steps above.

#### GC–MS analyses

Samples were analysed with a GC–MS QP 2010 Ultra system (Shimadzu, Japan). Chromatographic separation was performed on a Rxi-5 MS capillary column (30 m × 0.25 mm × 0.25 μm, RESTEK, USA). The GC oven temperature was set at 70°C for 2 minutes, then increased to 310°C at 5°C/minute increments and maintained at that temperature for 10 minutes. The carrier gas was helium (99.999%, China) and the linear velocity was 40 cm/second. The injection volume was 1 μL with a split ratio of 1:5. The temperatures of the injection, transfer line and ion source were 300, 280 and 230°C, respectively. An electron ionisation (EI) source was used. The detection voltage was set at 1.13 kV. Data acquisition started at 5.7 minutes and ended at 5.9 minutes, and the mass scan range was 33–600 m/z.

#### Metabolite identification

Metabolites were identified using an automated mass spectral deconvolution and identification system (AMDIS, NIST, USA) for spectral peak deconvolution and the National Institute of Standards and Technologies Mass Spectral library. A quantitative table containing the characteristic ions and the retention times of metabolites was imported to GC–MS Post run Analysis (Shimadzu, Japan) for batch processing of all samples. Identified metabolites with relative standard deviations < 10% in QC samples were used for further analyses. Multivariate analysis was conducted in SIMCA-P 11.5 (Umetrics, Sweden).

### IGF-1 analysis

Bound IGF-1 was separated from binding proteins by acid/ethanol (12.5% of 2 mol/L HCl and 87.5% ethanol) precipitation. Total IGF-1 levels were determined using an enzyme-linked immunoassay kit (Enzo, ADI 900-150). This assay was first validated for the Asian short-toed lark by serial plasma dilutions following the methods in Sparkman *et al*. (2009). Samples were assayed in duplicate. Mean intra-assay coefficients of variation were 5.2%, and the mean inter-assay coefficient of variation was 7.7%.

### Statistical analysis

**Figure 2:**
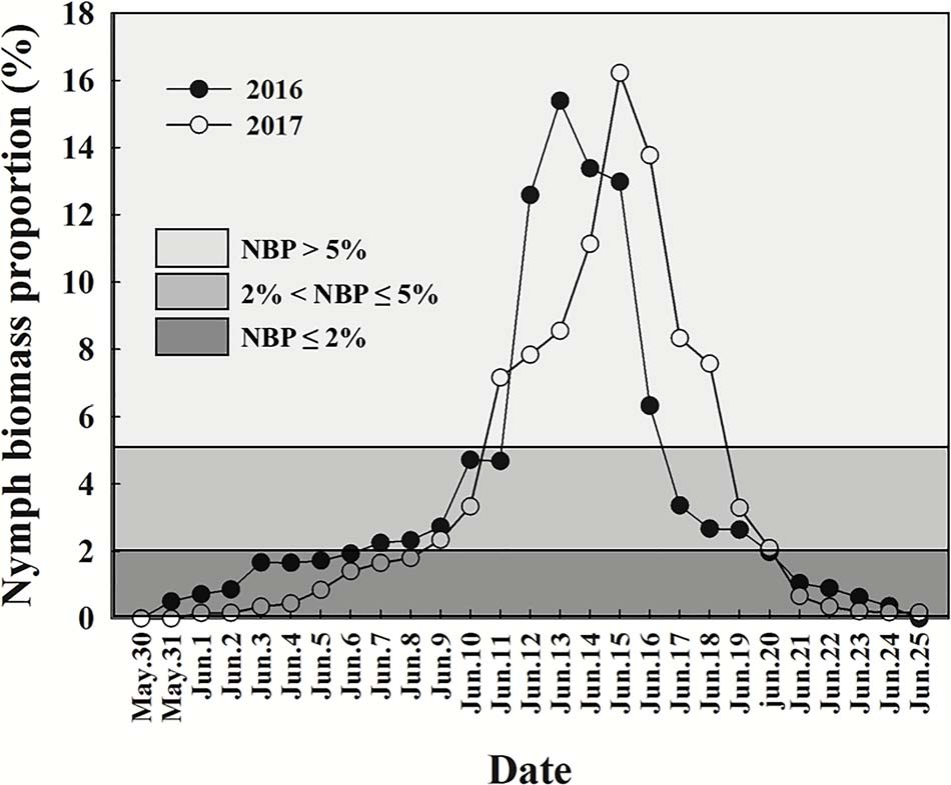
Temporal trend in grasshopper nymph biomass at Hulun lake nature reserve in 2016 and 2017. NBP is the percentage of the daily nymph biomass relative to the total biomass measured over all survey days. NBP > 5%, 2% < NBP ≤ 5% and NBP ≤ 2% represent high, medium and low nymph biomass, respectively.

We used “nymph biomass proportion (NBP)”, the percentage of the daily nymph biomass relative to the total biomass measured over all survey days, as a measure of grasshopper nymph abundance on different sampling days. Plotting NBP against date indicated that NBP peaked on the 13th and 15th of June in 2016 and 2017, respectively (Fig. 2). We observed an obvious, rapid increase in NBP when the NBP value reached 5%, a slower increase, or decrease, between 5 and 2%. NBP values < 2% were relatively stable (Fig. 2). Consequently, we categorized NBP as high (NBP > 5%), medium (2% < NBP ≤ 5%) and low (NBP ≤ 2%) (Fig. 2). Nestlings were assigned to these groups according to the date on which they reached four days of age, which ensured that nestlings remained in one of these NBP stages for at least half of the nestling period (the nestling period is 8 days). In 2016, 35, 25 and 24 nestlings were assigned into the NBP > 5%, 2% < NBP ≤ 5% and NBP ≤ 2% groups, respectively. In 2017, 30, 21 and 21 nestlings were assigned into the NBP > 5%, 2% < NBP ≤ 5% and NBP ≤ 2% groups, respectively.

All metabolite data were imported into SIMCA-P 11.5, and partial least-squares discriminant analysis (PLS-DA) was conducted to assess the differences of nestlings’ metabolites amongst three different NBP groups. Variable importance in the projection (VIP) in PLS-DA allowed the metabolites likely to be most responsible for between-group differences to be identified (Wone *et al*., 2011). Metabolites with VIP values > 1.0 in the model were regarded as potentially important and tested for statistical significance. Metabolic pathways for metabolites with significant between-group differences were obtained from the Kyoto Encyclopedia of Genes and Genomes (KEGG) database.

We tested the significance of differences in diet composition of nestlings amongst the three NBP groups with a two-way ANOVA. The statistical significance of inter-group differences in metabolite levels with VIP > 1.0, IGF-1 concentrations and body mass, of nestlings was assessed with a one-way ANOVA and Tukey’s HSD multiple comparison test. The strength and significance of correlations amongst metabolites, IGF-1 and body mass were examined using Pearson correlation. The survival rates of nestlings in three NBP groups were calculated as “number of nestlings hatched/number of nestlings fledging”, in which nestlings being preyed in the nest were not included in the “number of nestlings hatched”. All statistical analyses were performed in SPSS 22.0; *P*-values ≤ 0.05 were considered significant.

## Results

### Dietary differences amongst nestlings in different NBP groups

**Figure 3:**
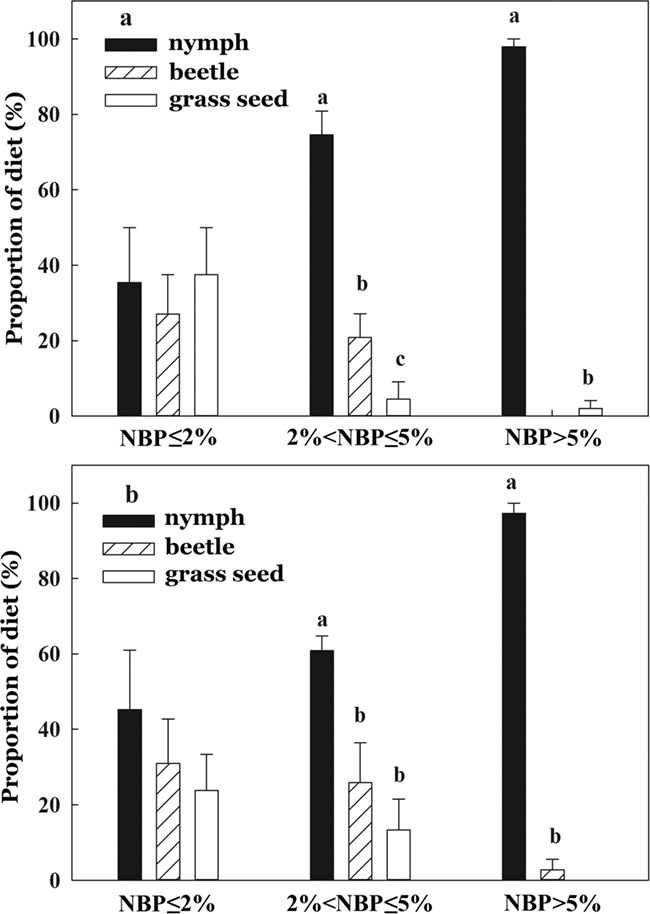
Composition of the diet of Asian short-toed lark (*C. cheleensis*) nestlings hatched during periods of high (NBP > 5%), moderate (2% < NBP ≤ 5%) and low (NBP ≤ 2%), grasshopper nymph abundance in (a) 2016 (*n* = 84) and (b) 2017 (*n* = 72).

The diets of nestlings in the high NBP group were mainly comprised of grasshopper nymphs, whereas those of nestlings in the medium or low NBP group were comprised of nymphs, beetles (coleoptera) and grass seeds (Fig. 3). The proportion of beetles and grass seeds in nestlings’ diets increased with decreasing NBP. The proportion of different foods (nymphs, beetles and grass seeds) in diets differed amongst the three NBP groups in both years (2016: Two-way ANOVA, diet composition × NBP group: *F*_(2, 81)_ = 18.12, *P* < 0.001; 2017: Two-way ANOVA, diet composition × NBP group *F*_(2, 69)_ = 12.63, *P* < 0.001, Fig. 3). The proportion of nymphs in diets of nestlings in the high NBP group was significantly higher than in the other two groups (2016: One-way ANOVA, *F*_(2,81)_ = 11.92, *P* < 0.001, Tukey’s HSD test, all *P* < 0.05; 2017: One-way ANOVA, *F*_(2, 69)_ = 10.64, *P* < 0.001, Tukey’s HSD test, all *P* < 0.05) (Fig. 3).

### Metabolite differences amongst nestlings in different NBP groups

**Figure 4:**
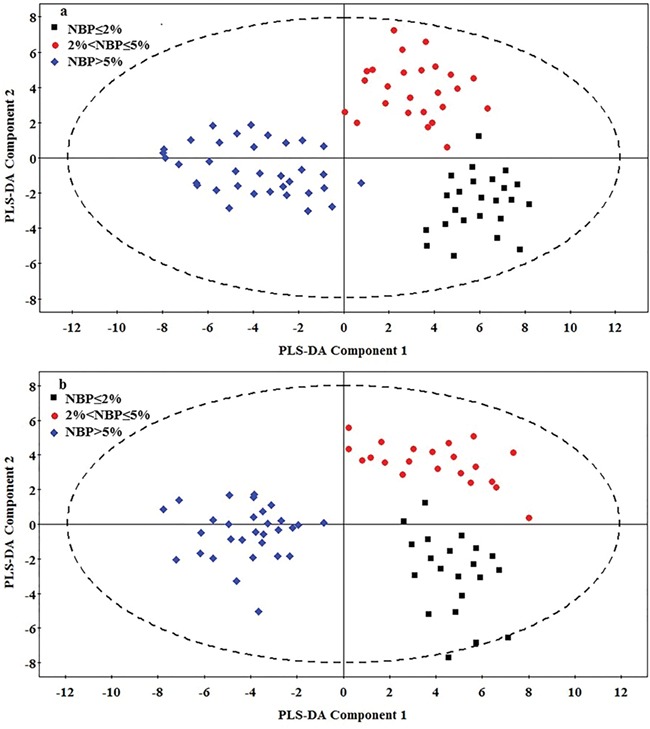
PLS-DA score plot of serum metabolic profiles of Asian short-toed Lark (*C. cheleensis*) nestlings hatched during periods of high (>5%), moderate (between 2 and 5%) and low (≤ 2%) grasshopper nymph abundance (NBP) in 2016 (a) and 2017 (b).

In 2016, the PLS-DA model explained 76.10% of the variance (*R*^2^*Y*) and predicted 64.50% (*Q*^2^*Y*) of the dataset (Fig. 4a). In 2017, the model explained 77.33% of the variance (*R*^2^*Y*) and predicted 66.32% (*Q*^2^*Y*) of the dataset (Fig. 4b). Therefore, the model revealed differences in serum metabolomics amongst nestlings in the three NBP groups in both years of the study. Metabolite data from nestlings clustered according to their NBP group (Fig. 4). A one-way ANOVA indicates that 29 metabolites with VIP values > 1 were significantly different amongst the three NBP groups in both 2016 (Table 1) and 2017 (Table 2). According to the metabolite functions obtained from KEGG, these metabolites are tricarboxylic acid (TCA) cycle metabolites, glucides, fatty acids and amino acids. Multiple comparison tests for each metabolite indicate the glucides, amino acids, TCA cycle metabolites and most fatty acids of the high NBP group were significantly higher than those of the other two groups (Tukey’s HSD test, *P* < 0.05), whereas four fatty acids, including palmitoleic acid, oleic acid, linoleic acid and arachidic acid, were significantly higher in the medium and low NBP groups (Tukey’s HSD test, *P* < 0.05) (Tables 1 and 2).

**Table 1:** Relative abundance of 29 metabolites with VIP values > 1.0 in a PLS-DA model in plasma of Asian Short-toed Lark (*C. cheleensis*) nestlings in 2016 (*n* = 84). Different letters (a, b, c) above values indicate significant between-group differences as assessed by an ANOVA followed by Tukey’s multiple comparison test

Metabolites		NBP ≤ 2%	2% < NBP ≤ 5%	NBP > 5%	*F*	*P*
TCA	α-Ketoglutaric acid	0.25 ± 0.02 ^a^	0.33 ± 0.06 ^b^	0.35 ± 0.078 ^b^	8.19	< 0.01
	Fumaric acid	0.42 ± 0.07 ^a^	0.69 ± 0.04 ^b^	0.80 ± 0.04 ^b^	11.19	< 0.01
	Malic acid	0.46 ± 0.03 ^a^	0.47 ± 0.03 ^a^	0.61 ± 0.11 ^b^	6.18	< 0.01
	Succinic acid	0.69 ± 0.07 ^a^	0.66 ± 0.06 ^a^	0.97 ± 0.08 ^b^	5.01	< 0.05
Glucide	Glucose	2.73 ± 0.48 ^a^	3.24 ± 0.77 ^a^	4.44 ± 0.98 ^b^	9.10	< 0.01
	Turanose	0.73 ± 0.04 ^a^	0.80 ± 0.03 ^a^	0.90 ± 0.03 ^b^	6.90	< 0.01
	Fructose 6 phosphate	0.21 ± 0.02 ^a^	0.25 ± 0.02 ^a^	0.26 ± 0.10 ^b^	4.56	< 0.01
Fatty acids	Cholesterol	1.48 ± 0.09 ^a^	1.64 ± 0.07 ^b^	1.87 ± 0.08 ^b^	5.53	< 0.01
	Monopalmitin	0.043 ± 0.002 ^a^	0.049 ± 0.002 ^a^	0.055 ± 0.002^b^	9.82	< 0.01
	14:0 Fatty acid	0.18 ± 0.01 ^a^	0.25 ± 0.02 ^a^	0.32 ± 0.02 ^b^	6.71	< 0.01
	16:0 Fatty acid	0.06 ± 0.02 ^a^	0.09 ± 0.02 ^ab^	0.12 ± 0.02 ^b^	4.56	< 0.01
	16:1 Fatty acid	0.54 ± 0.07 ^b^	0.43 ± 0.05 ^b^	0.15 ± 0.03 ^a^	7.95	< 0.01
	18:1 Fatty acid	0.28 ± 0.01 ^a^	0.27 ± 0.02 ^a^	0.21 ± 0.02 ^b^	5.37	< 0.01
	20:0 Fatty acid	0.14 ± 0.01 ^a^	0.14 ± 0.04 ^a^	0.12 ± 0.04 ^b^	5.52	< 0.01
	20:4 Fatty acid	0.38 ± 0.05 ^a^	0.57 ± 0.03 ^b^	0.59 ± 0.04 ^b^	6.23	< 0.01
	22:6 Fatty acid	0.18 ± 0.02 ^a^	0.29 ± 0.03 ^b^	0.32 ± 0.01 ^b^	7.90	< 0.01
Amino acids	Alanine	1.22 ± 0.07 ^a^	1.37 ± 0.07 ^a^	1.69 ± 0.11 ^b^	5.50	< 0.01
	Aspartic acid	0.93 ± 0.12 ^a^	0.95 ± 0.11 ^a^	1.56 ± 0.10 ^b^	11.09	< 0.01
	Asparagine	0.31 ± 0.02 ^a^	0.41 ± 0.07 ^a^	0.66 ± 0.07 ^b^	7.43	< 0.01
	Hydroxyproline	0.66 ± 0.08 ^ab^	0.44 ± 0.04 ^a^	0.75 ± 0.07 ^b^	5.91	< 0.01
	Isoleucine	1.07 ± 0.07 ^a^	1.31 ± 0.07 ^a^	1.64 ± 0.12 ^b^	5.97	< 0.01
	Leucine	1.49 ± 0.10 ^a^	1.92 ± 0.11 ^a^	2.99 ± 0.13 ^b^	8.54	< 0.01
	Methionine	0.54 ± 0.04 ^a^	0.61 ± 0.04 ^a^	0.81 ± 0.05 ^b^	8.58	< 0.01
	Proline	2.54 ± 0.06 ^a^	2.66 ± 0.11 ^a^	3.14 ± 0.16 ^b^	5.01	< 0.05
	Phenylalanine	0.72 ± 0.13 ^a^	0.79 ± 0.14 ^a^	1.63 ± 0.21 ^b^	10.61	< 0.01
	Serine	1.56 ± 0.10 ^a^	1.86 ± 0.10 ^b^	1.95 ± 0.06 ^b^	4.76	< 0.05
	Threonine	0.75 ± 0.12 ^a^	1.10 ± 0.07 ^a^	1.56 ± 0.17 ^b^	6.25	< 0.01
	Tyrosine	0.62 ± 0.04 ^a^	0.69 ± 0.05 ^a^	1.11 ± 0.07 ^b^	14.70	< 0.01
	Valine	1.78 ± 0.12 ^a^	1.92 ± 0.11 ^a^	2.74 ± 0.16 ^b^	13.16	< 0.01

**Table 2:** Relative abundance of 29 metabolites with VIP values > 1.0 in a PLS-DA model in plasma of Asian Short-toed Lark (*C. cheleensis*) nestlings in 2017 (*n* = 72). Different letters (a, b, c) above values indicate significant between-group differences as assessed by an ANOVA followed by Tukey’s multiple comparison test

Metabolites		NBP ≤ 2%	2% < NBP ≤ 5%	NBP > 5%	*F*	*P*
TCA	Citric acid	0.40 ± 0.10 ^a^	0.46 ± 0.16 ^ab^	0.54 ± 0.18 ^b^	3.73	< 0.05
	Malic acid	0.38 ± 0.08 ^a^	0.38 ± 0.13 ^a^	0.53 ± 0.19 ^b^	6.26	< 0.01
	Succinic acid	0.95 ± 0.13 ^a^	1.47 ± 0.17 ^b^	1.91 ± 0.15 ^c^	9.11	< 0.01
Glucide	Glucose	5.87 ± 0.24 ^a^	5.51 ± 0.24 ^a^	6.65 ± 0.23 ^b^	6.17	< 0.01
	Trehalose	2.37 ± 0.47 ^a^	2.39 ± 0.60 ^a^	3.09 ± 0.93 ^b^	6.26	< 0.01
Fatty acids	Cholesterol	1.21 ± 0.23 ^a^	1.45 ± 0.29 ^ab^	1.77 ± 0.18 ^b^	4.50	< 0.05
	Glyceryl myrisate	0.053 ± 0.014 ^a^	0.058 ± 0.015 ^a^	0.074 ± 0.017 ^b^	11.41	< 0.01
	Glyceryl palmitate	0.039 ± 0.003 ^a^	0.043 ± 0.003 ^a^	0.054 ± 0.002 ^b^	15.75	< 0.01
	Vitamin E	0.65 ± 0.14 ^a^	0.73 ± 0.12 ^a^	0.91 ± 0.23 ^b^	9.65	< 0.01
	16:0 Fatty acid	0.14 ± 0.04 ^a^	0.14 ± 0.03 ^a^	0.17 ± 0.03 ^b^	5.70	< 0.01
	18:0 Fatty acid	0.48 ± 0.04 ^a^	0.56 ± 0.09 ^a^	0.63 ± 0.09 ^b^	11.32	< 0.01
	18:1 Fatty acid	0.20 ± 0.02 ^a^	0.18 ± 0.02 ^a^	0.12 ± 0.01 ^b^	7.81	< 0.01
	18:2 Fatty acid	0.90 ± 0.12 ^a^	0.79 ± 0.03 ^ab^	0.51 ± 0.03 ^b^	11.69	< 0.01
	20:0 Fatty acid	0.10 ± 0.03 ^a^	0.10 ± 0.04 ^a^	0.07 ± 0.02 ^b^	9.07	< 0.01
	20:5 Fatty acid	0.26 ± 0.02 ^a^	0.31 ± 0.02 ^a^	0.41 ± 0.03 ^b^	9.18	< 0.01
Amino acids	Alanine	1.41 ± 0.13 ^a^	1.38 ± 0.09 ^a^	1.94 ± 0.15 ^b^	4.75	< 0.05
	Aspartic acid	1.07 ± 0.12 ^a^	1.71 ± 0.13 ^b^	2.25 ± 0.17 ^c^	11.64	< 0.01
	Asparagine	0.35 ± 0.14 ^a^	0.53 ± 0.07 ^a^	0.90 ± 0.09 ^b^	3.37	< 0.05
	Hydroxyproline	0.56 ± 0.07 ^ab^	0.45 ± 0.01 ^a^	0.78 ± 0.02 ^b^	6.09	< 0.01
	Isoleucine	1.48 ± 0.22 ^a^	2.04 ± 0.13 ^a^	2.57 ± 0.24 ^b^	5.07	< 0.01
	Leucine	1.32 ± 0.35 ^a^	1.68 ± 0.20 ^a^	3.25 ± 0.51 ^b^	4.64	< 0.01
	Lysine	0.54 ± 0.07 ^a^	0.85 ± 0.01 ^ab^	1.06 ± 0.16 ^b^	5.00	< 0.01
	Methionine	0.59 ± 0.24 ^a^	0.82 ± 0.26 ^ab^	0.98 ± 0.35 ^b^	7.01	< 0.01
	Proline	1.88 ± 0.17 ^a^	2.26 ± 0.18 ^ab^	2.88 ± 0.23 ^b^	4.77	<0.05
	Phenylalanine	0.80 ± 0.09 ^a^	1.07 ± 0.07 ^ab^	1.19 ± 0.08 ^b^	4.54	< 0.05
	Serine	1.43 ± 0.26 ^a^	1.66 ± 0.39 ^ab^	1.97 ± 0.64 ^b^	5.13	< 0.01
	Threonine	0.91 ± 0.10 ^a^	1.13 ± 0.10 ^a^	2.01 ± 0.26 ^b^	6.28	< 0.01
	Tyrosine	0.69 ± 0.05 ^a^	0.86 ± 0.10 ^ab^	1.07 ± 0.09 ^b^	5.70	< 0.01
	Valine	1.36 ± 0.32 ^a^	1.49 ± 0.19 ^a^	1.74 ± 0.32 ^b^	5.01	< 0.05

### IGF-1, body mass and survival rate differences amongst nestlings in different NBP groups

**Figure 5:**
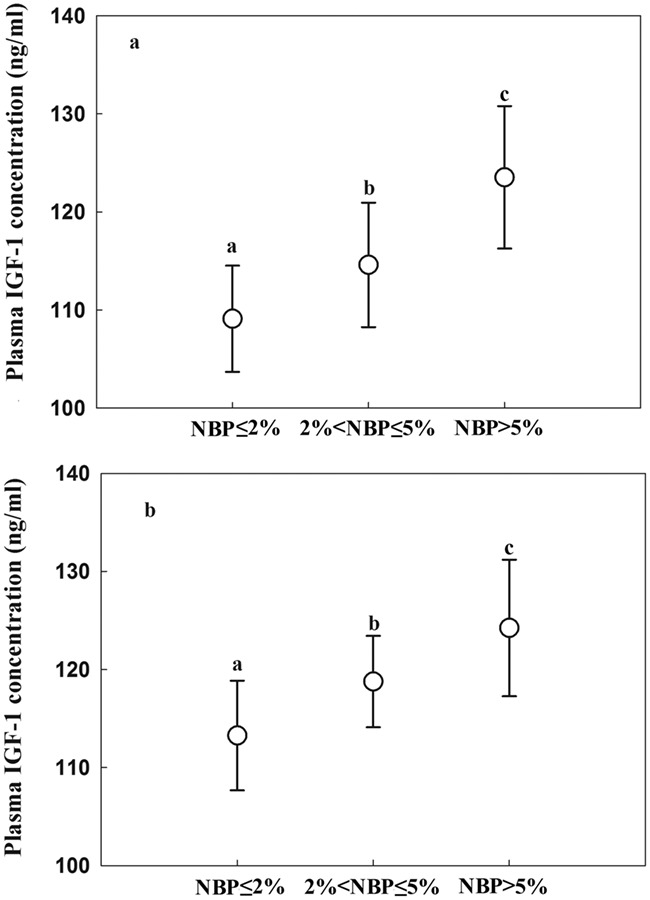
Plasma IGF-1 concentrations of Asian short-toed lark (*C. cheleensis*) nestlings hatched during periods of high (NBP > 5%), moderate (2% < NBP ≤ 5%), and low (NBP ≤ 2%), grasshopper nymph abundance in (a) 2016 (*n* = 84) and (b) 2017 (*n* = 72). Different letters above error bars indicate significant between-group differences as assessed by an ANOVA followed by Tukey’s multiple comparison test.

**Figure 6:**
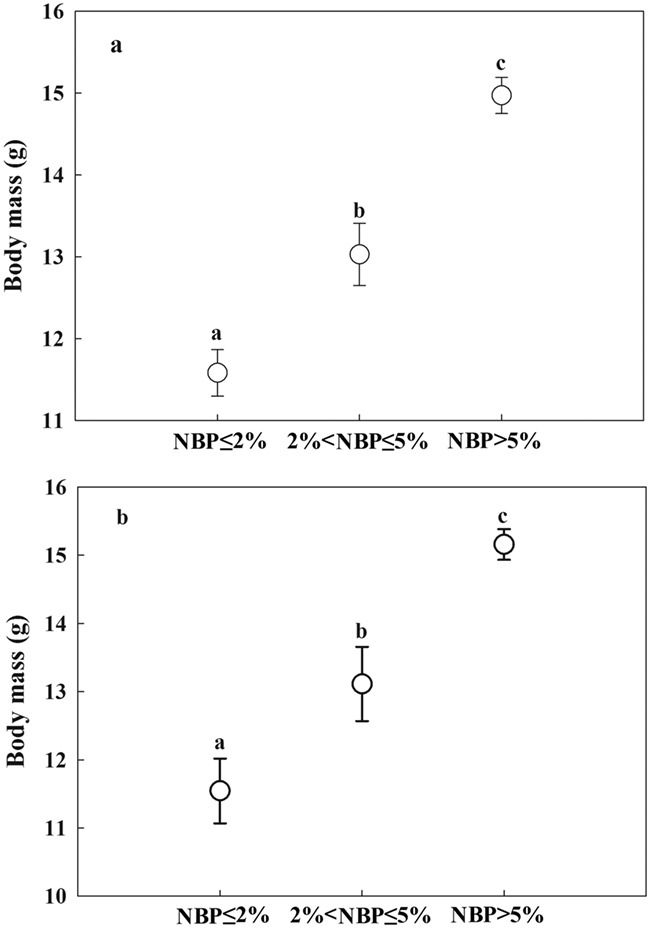
Body mass of Asian short-toed lark (*C. cheleensis*) nestlings hatched during periods of high (NBP > 5%), moderate (2% < NBP ≤ 5%) and low (NBP ≤ 2%), grasshopper nymph abundance in (a) 2016 (*n* = 84) and (b) 2017 (*n* = 72). Different letters above error bars indicate significant between-group differences as assessed by an ANOVA followed by Tukey’s multiple comparison test.

The three NBP groups differed significantly in plasma IGF-1 levels (2016: one-way ANOVA, *F*_(2, 81)_ = 18.02, *P* < 0.001; 2017: one-way ANOVA, *F*_(2, 69)_ = 17.92, *P* < 0.001). Plasma IGF-1 in the high NBP group was significantly higher than in the medium or low groups (Tukey’s HSD test, *P* < 0.05), but there was no significant difference in plasma IGF-1 between the medium and low groups (Tukey’s HSD test, *P* > 0.05) (Fig. 5). Body mass also differed significantly amongst the three NBP groups (2016: one-way ANOVA, *F*_(2, 81)_ = 56.25, *P* < 0.001; 2017: one-way ANOVA, *F*_(2, 69)_ = 51.83, *P* < 0.001); nestlings in the high NBP group had the highest body mass and those in the low NBP group had the lowest (Tukey’s HSD test, *P* < 0.05) (Fig. 6). In 2016, the nestling survival rate were 66.7, 83.3 and 92.0% in low, medium and high NBP groups, respectively. In 2017, the nestling survival rates were 75, 88.89 and 96.6% in low, medium and high NBP groups, respectively.

### Correlations between plasma metabolites, plasma IGF-1 and nestling body mass

Plasma IGF-1 level was positively correlated with body mass (2016: *r* = 0.88, *P* < 0.001; 2017: *r* = 0.89, *P* < 0.001) and with the mean relative abundance of serum TCA cycle metabolites (2016: *r* = 0.83, *P* < 0.001; 2017: *r* = 0.89, *P* < 0.001), glucides (2016: *r* = 0.92, *P* < 0.001; 2017: *r* = 0.91, *P* < 0.001), fatty acids (2016: *r* = 0.73, *P* < 0.001; 2017: *r* = 0.76, *P* < 0.001) and amino acids (2016: *r* = 0.87, *P* < 0.001; 2017: *r* = 0.82, *P* < 0.001).

## Discussion

Our results show that the proportion of grasshopper nymphs in the diet of Asian short-toed lark nestlings decreased in two successive years when the NBP was < 5%, and that under such conditions parent birds supplemented the diet of nestlings with beetles and grass seeds. When the NBP was < 2%, there was no significant difference in the proportion of grass seeds, beetles and grasshopper nymphs provided to nestlings. These results indicate that Asian short-toed larks attempt to compensate for lower grasshopper nymph abundance by providing nestlings with alternate foods. This supports our first hypothesis that alternative foods are provided to nestlings when the optimal food is not abundant. This result is consistent with those obtained on other species, such as the great tit (*Parus major*) (Naef-Daenzer *et al*., 2000; Wilkin *et al*., 2009), blue tit (*Parus caeruleus*) (Grieco, 2001; Tremblay *et al*., 2005; García-Navas and Sanz, 2011), wood warbler (*Phylloscopus sibilatrix*) (Maziarz and Wesołowski, 2010) and pied flycatcher (*Ficedula hypoleuca*) (Samplonius *et al*., 2016). This suggests that bird species attempt to compensate for the phenological mismatch by providing alternate foods to nestlings. However, in most of available studies, it was not clear if the alternative foods had the same nutritional quality as the optimal food. Assessing the benefit of such adjustments requires measuring nutrients actually absorbed by nestlings from different diets rather than just quantifying dietary components.

Our metabolomic analysis indicates that nestlings that were fed more beetles and grass seeds absorbed significantly less amino acids from diets than those that were mostly fed nymphs. Amino acids are the basic units of protein and are therefore essential for protein synthesis during nestling development (Wu, 2010). Essential amino acids (EAAs) are not synthesized by animal cells and therefore must be obtained from diets (Wu, 2010). Therefore, the amount of amino acids, and especially EAAs, obtained from food directly affects the growth and development of young animals (Ramsay and Houston, 2003; Wu, 2010). We found that nine EAAs (valine, poline, hydroxyproline, leucine, threonine, isoleucine, methionine, phenyllamine and lysine) were significantly lower in nestlings in the medium and low NBP groups, which suggest that the alternative foods provided to these nestlings were not as high in EAAs as grasshopper nymphs. It is possible that the protein synthesis of nestlings in these groups could be limited by their alternative diets. Grasshopper nymphs and beetles contain 62.4–67.3% and 41.8–51.6% protein, respectively (Ye *et al*., 1998 ; Sun *et al*., 2011), which might explain why the amino acid levels of nestlings in the high NBP group exceeded that of the other two groups. However, a study on one population of blue tits found that the caterpillars that comprised the majority of chicks’ diets throughout the rearing period had lower protein content than the coleoptera that were also fed to nestlings (Ramsay and Houston, 2003). Such findings suggest that protein content alone may not be a good indicator of a food’s nutritional quality. Consequently, quantifying the plasma nutritional status of nestlings is a more reliable way of determining the nutritional quality of their diets than determining the nutritional content of their food.

Except for four unsaturated fatty acids, nestlings fed a higher proportion of beetles and grass seeds absorbed significantly lower amounts of fatty acids and glucides than those fed predominantly on grasshopper nymphs. Glucides and fatty acids are substrates of the energy metabolism (Rodgers and Puigserver, 2007). The lower levels of these compounds in nestlings in the low NBP group suggest that the alternative foods provided to these nestlings less energy substrates than grasshopper nymphs did. The TCA cycle is the most important metabolic pathway of energy generation in animal cells (McCammon *et al*., 2003) and the amount of blood metabolites involved in the TCA cycle pathway is positively related to the level of energy production in the whole body (McCammon *et al*., 2003). The lower levels of serum energy substrates and TCA metabolites in the low NBP group suggest that alternative foods provide lower levels of energy than grasshopper nymphs.

Growth is an energetically and nutritionally demanding processes (Kitaysky 1999; van der Ziel and Visser, 2001) and increased nutrition and energy intake have been found to sustain the levels of IGF-1 secretion required to mediate higher growth rates (Ross and Buchanan, 1990; Breier, 1999). IGF-1 levels are correlated with those of various metabolic compounds, including glucose, insulin, free fatty acids and amino acids (Ross and Buchanan, 1990; Scacchi *et al*., 2003). Good nutrition can therefore promote IGF-1 synthesis (Ross and Buchanan, 1990; Mohan and Kesavan, 2012), whereas poor nutrition causes IGF-1 levels to decrease (Breier *et al*., 1999; Scacchi *et al*., 2003). Our results that nestlings in the medium and low NBP groups had significantly lower plasma IGF-1 levels and body mass than those in the high NBP group indicate that the lower blood nutrients can result in lower growth hormone and lower body condition. The positive correlation between plasma IGF-1 levels and body mass suggests that the alternative food items provided to these nestlings caused them to have lower body condition than nestlings in the high NBP group. Low body condition could finally result in the low fitness in adult period (e.g. Golet, 2000).

Climate change induced phenological mismatches between nestlings and peaks of optimal food abundance are likely to have a greater impact on the survival of bird populations (Visser *et al*., 1998; Ovaskainen *et al*., 2013; Doiron *et al*., 2015). The results of nestling plasma metabolism and survival rate in this study suggest that the diet with low proportion optimal food could result in low viability of nestlings mediated by malnutrition. Energy stress induced AMP-activated protein kinase (AMPK)-apoptosis pathway could be related with the food quality and survival of nestlings. Plasma AMPK is a primary regulator of the cellular response to lowered energy (ATP) levels in eukaryotic cells (Hardie *et al*., 2003) and the activation of AMPK is associated with activation of caspase-3 to induce apoptosis (Kefas *et al*., 2003). We have found that the Asian short-toed lark population we studied experienced trophic mismatch in 2014 and 2016 caused by extreme spring temperature, which resulted in remarkable nestling survival rate decline in those 2 years (Zhang *et al*., 2017). Therefore, under the climate change scenario, the species depending on one type of optimal food like Asian short-toed lark could encounter the crisis of population decline, because the adults were not able to correctly adjust their timing of breeding to track the shift of optimal food abundance peak and adjusting diets of nestlings could also be weak to mitigate this negative effect. We suggest that a species’ vulnerability to a phenological mismatch should not only be assessed by observation of its diet in the field but also by measurement of nestlings’ nutritional status and body condition. In this article, we provide a case to predict the vulnerability of species to the phenological mismatch induced trophic threat. If the nutritional status and body condition of nestlings fed alternative foods are same as those of nestlings that are predominantly fed the optimal food, the population is probably not adversely affected by the phenological mismatch. However, if that is not the case, then the population concerned could be affected, possibly to the extent that it declines. Therefore, predicting the vulnerability of bird species to phenological mismatches requires investigation of the nutritional quality of nestlings’ diets, their nutritional status and body condition.
